# The Significance of an Initial Controlling Nutritional Status Score in Predicting the Functional Outcome, Complications, and Mortality in a First-Ever Ischemic Stroke

**DOI:** 10.3390/nu16203461

**Published:** 2024-10-12

**Authors:** Hyoseon Choi, Yea Jin Jo, Min Kyun Sohn, Jongmin Lee, Yong-Il Shin, Gyung-Jae Oh, Yang-Soo Lee, Min Cheol Joo, So Young Lee, Min-Keun Song, Junhee Han, Jeonghoon Ahn, Young-Hoon Lee, Yun-Hee Kim, Won Hyuk Chang, Deog Young Kim

**Affiliations:** 1 Department of Rehabilitation Medicine, Nowon Eulji Medical Center, Eulji University, Seoul 01830, Republic of Korea; 0703hs@gmail.com; 2 Department of Rehabilitation Medicine, Yonsei University College of Medicine, Seoul 03722, Republic of Korea; 3 Department of Exercise Rehabilitation & Welfare, Gachon University, 191, Hambangmore-ro, Yeonsu-gu, Incheon 21936, Republic of Korea; yejn842@gachon.ac.kr; 4 Department of Rehabilitation Medicine, College of Medicine, Chungnam National University, Daejeon 34134, Republic of Korea; mksohn@cnu.ac.kr; 5 Department of Rehabilitation Medicine, Konkuk University School of Medicine, Seoul 05030, Republic of Korea; leej@kuh.ac.kr; 6 Department of Rehabilitation Medicine, Pusan National University School of Medicine, Pusan National University Yangsan Hospital, Yangsan 50612, Republic of Korea; rmshin01@gmail.com; 7 Department of Preventive Medicine, School of Medicine, Wonkwang University, Iksan 54538, Republic of Korea; pmokj@wku.ac.kr (G.-J.O.); lyh8275@hanmail.net (Y.-H.L.); 8 Department of Rehabilitation Medicine, School of Medicine, Kyungpook National University, Kyungpook National University Hospital, Daegu 41944, Republic of Korea; leeyangsoo@knu.ac.kr; 9 Department of Rehabilitation Medicine, Wonkwang University School of Medicine, Iksan 54538, Republic of Korea; jmc77@hanmail.net; 10 Department of Rehabilitation Medicine, Jeju National University Hospital, Jeju National University School of Medicine, Jeju 63241, Republic of Korea; bluelsy900@hanmail.net; 11 Department of Physical and Rehabilitation Medicine, Chonnam National University Medical School, Gwangju 61469, Republic of Korea; drsongmk@daum.net; 12 Division of Data Science and Data Science Convergence Research Center, Hallym University, Chuncheon 24252, Republic of Korea; pnuyh.rass@gmail.com; 13 Department of Health Convergence, Ewha Womans University, Seoul 03760, Republic of Korea; ahnjeonghoon@ewha.ac.kr; 14 Department of Physical and Rehabilitation Medicine, Sungkyunkwan University School of Medicine, Suwon 16419, Republic of Korea; yunkim@skku.edu; 15 Department of Physical and Rehabilitation Medicine, Center for Prevention and Rehabilitation, Heart Vascular Stroke Institute, Samsung Medical Center, Sungkyunkwan University School of Medicine, Seoul 06351, Republic of Korea; 16 Department of Health Science and Technology, Department of Medical Device Management and Research, Department of Digital Healthcare, SAIHST, Sungkyunkwan University, Seoul 06351, Republic of Korea; 17 Research Institute of Rehabilitation Medicine, Yonsei University College of Medicine, Seoul 03722, Republic of Korea

**Keywords:** stroke, cerebral infarction, nutritional status, functional recovery, survival

## Abstract

Background and Purpose: Nutritional status can influence the outcomes and mortality of various diseases. The association between initial nutritional status and ischemic stroke outcomes, however, remains poorly understood. This study investigated whether the Controlling Nutritional Status (CONUT) score at admission could predict functional recovery, complications, and survival following an ischemic stroke. Methods: We enrolled a total of 938 patients experiencing their first acute ischemic stroke and categorized them into three groups based on their CONUT score at admission: CONUT 0–1, CONUT 2–4, and CONUT 5–12. The CONUT score was assessed using the serum albumin, total cholesterol, and lymphocyte count. We evaluated the incidence of complications during their hospital stay. Outcomes, including the Modified Rankin Scale (mRS), Functional Independence Measurement (FIM), Functional Ambulatory Classification (FAC), and mortality, were assessed at baseline, as well as at three and six months post-stroke. Results: CONUT scores were significantly associated with functional outcomes (mRS, FIM, and FAC) and mortality during the six-month follow-up period post-stroke (all *p* < 0.05). The CONUT 5–12 group exhibited significantly poorer improvements in mRS, FIM, and FAC scores (all *p* < 0.05) and a lower survival rate (*p* < 0.01) during the six-month follow-up compared to the CONUT 0–1 and CONUT 2–4 groups. Additionally, the incidence of pneumonia, urinary tract infections, pressure sores, falling injuries, and fractures was significantly higher in the CONUT 5–12 group than in the other groups (all *p* < 0.01). Conclusions: CONUT scores at admission are associated with functional recovery, mortality, and the incidence of complications following a first-ever ischemic stroke. Consequently, the early identification of patients at risk of malnutrition via CONUT scores can be crucial in enhancing patient assessment after an acute stroke.

## 1. Introduction

Malnutrition is characterized by inadequate or imbalanced nutrient intake, which leads to or exacerbates body composition abnormalities [1]. As a significant public health issue, malnutrition is associated with poorer prognoses in medical, geriatric, and surgical patients [2,3]. Malnourished patients are more likely to be hospitalized, experience more procedure-related complications, and face higher mortality risks compared to their well-nourished counterparts [4,5]. The prevalence of malnutrition among stroke hospitalizations can be as high as 62% [6]. Additionally, between 16 and 49% of patients with acute stroke are malnourished upon admission [7,8]. Despite its high prevalence, the impact of nutritional status on functional recovery in stroke patients has not been extensively explored.

Recent guidelines have advocated for the use of nutritional support in patients with or at risk of malnutrition [9]. Nevertheless, the effects of initial nutritional status on functional outcomes and survival rates in stroke remain ambiguous. Previous studies on nutritional status and functional outcomes in acute stroke have yielded inconsistent results—some suggest that malnutrition leads to poor clinical outcomes [8,10,11], while others report conflicting findings [7,12].

The Controlling Nutritional Status (CONUT) scoring system, developed as a straightforward screening tool, facilitates the early detection of poor nutritional status [13]. It utilizes simple objective markers such as serum albumin levels, total cholesterol concentration, and total lymphocyte count, which enable a cost-effective and objective evaluation of nutritional status in acute clinical settings [13]. The CONUT score has proven to be a reliable tool for the early detection and ongoing control of malnutrition in hospital settings. A previous study comparing the CONUT score with other nutrition-related tools, including NRS-2002, the Onodera Prognostic Nutritional Index (OPNI), the Instant Nutritional Assessment, and the Geriatric Nutritional Risk Index (GNRI), found that the CONUT score demonstrated the highest predictive ability for in-hospital mortality in older adult patients, suggesting superior predictive validity [14]. It has been linked with outcomes in cancer, heart disease, and liver disease [15,16,17]. Compared to simpler evaluation tools like the PNI, which only measures albumin and lymphocytes, the CONUT score requires additional testing but provides a more comprehensive assessment of nutritional status by including cholesterol levels. Although it does not evaluate components such as BMI, muscle mass, and weight loss, as included in the Global Leadership Initiative on Malnutrition criteria, its strength lies in offering a quick and easy lab-based evaluation. This makes it particularly valuable in clinical settings, especially for hospitalized stroke patients requiring urgent treatment [18,19].

However, reports on its application in patients with ischemic stroke are scant [20,21]. Limited data exist concerning differences in functional outcomes based on this scoring system. Understanding whether CONUT scores can predict functional outcomes following ischemic stroke may provide further evidence and clearer clinical implications for the importance of early identification of patients at risk of malnutrition. Therefore, this study aims to explore the associations between nutritional status, assessed using CONUT scores at admission, and the functional outcomes and survival rates in patients with ischemic stroke.

## 2. Methods

### 2.1. Study Cohort

Patients who experienced an ischemic stroke and were recruited for the Korean Stroke Cohort for Functioning and Rehabilitation (KOSCO) study at Severance Hospital were included in this analysis. The KOSCO study is a prospective, 10-year follow-up cohort investigation of patients with acute first-time strokes who were admitted to representative Korean hospitals from August 2012 to March 2015. This study aimed to explore the factors that influence residual disabilities and activity limitations. The selection procedure for patients and protocols for the KOSCO study have been thoroughly documented previously [19]. The inclusion criteria were as follows: (1) diagnosis of a first-ever ischemic stroke as confirmed by brain computed tomography (CT) or magnetic resonance imaging (MRI); (2) age of 19 years or older at the time of stroke onset; and (3) presentation of symptoms within seven days prior to hospital admission. The exclusion criteria included the following: (1) individuals with a history of recurrent stroke; (2) patients diagnosed with a transient ischemic attack; (3) those who declined to provide informed consent; and (4) patients who were lost to follow-up.

Written informed consent was obtained from all participants or their guardians. This study received approval from the Ethics Committee of the Institutional Review Board (4-2012-0341) and was conducted in accordance with the principles of the Declaration of Helsinki.

### 2.2. Risk Factors and Nutritional Assessment

Stroke risk factors and comorbidities were meticulously recorded, covering age, sex, hypertension, diabetes mellitus, coronary heart disease, atrial fibrillation, hyperlipidemia, as well as historical data on smoking and alcohol consumption, and the presence of heart, renal, liver, or malignant diseases: hypertension (systolic blood pressure > 160 mmHg, diastolic blood pressure > 90 mmHg, or history of hypertension or medical treatment); diabetes mellitus (elevated blood glucose level > 126 mg/day or history of diabetes or medical treatment); coronary heart disease (documented by standard ECG or coronary imaging study or history of coronary heart disease or medical treatment); atrial fibrillation (documented by standard ECG, long-term ECG, or history of atrial fibrillation or medical treatment); and hyperlipidemia (elevated LDL cholesterol level > 160 mg/dL, elevated total cholesterol level > 240 mg/dL, or history of hyperlipidemia or medical treatment) [22]. Body mass index (BMI) was determined by measuring weight and height. The initial blood tests upon admission included evaluations of albumin, total cholesterol, total lymphocyte count, and *C*-reactive protein (CRP).

The nutritional status of patients was assessed using the CONUT score immediately after admission. The CONUT score is a tool for estimating nutritional status that evaluates protein reserves, calorie depletion, and immune defenses [23]. It incorporates three sub-scores based on the following: (1) serum albumin concentration, reflecting protein reserves [24,25]; (2) total cholesterol level, important in steroid biosynthesis; and (3) total lymphocyte count, indicative of immune function and typically diminished during nutritional depletion. The aggregate score ranges from 0 (indicating no malnutrition) to 12 (reflecting severe malnutrition) [2,3]. Based on this scoring system, patients were stratified into three categories: CONUT 0–1, CONUT 2–4, and CONUT 5–12.

### 2.3. Outcome Measures

Stroke severity was assessed using the NIHSS upon admission. The completion of acute neurological management was defined by either discharge from the neurology department or transfer to the intensive rehabilitation ward upon achieving neurological stability. At this juncture, functional status was evaluated using three standardized tools: the modified Rankin Scale (mRS) [26], the Functional Independence Measurement (FIM) [27], and the Functional Ambulatory Classification (FAC) [28]. Additionally, functional status was reassessed at both three and six months post-stroke. During the hospital stay, complications such as thromboembolic disease, pneumonia, ventilatory insufficiency, urinary tract infections, pressure sores, injurious falls, fractures, complex regional pain syndrome, and central post-stroke pain syndrome were meticulously documented.

### 2.4. Statistical Analysis

A Fisher’s exact or χ^2^ test was employed to compare the frequencies of categorical variables across groups. A Kolmogorov–Smirnov test assessed the normality of continuous variables. Depending on their distribution, differences in continuous variables were analyzed using either a Kruskal–Wallis test or ANOVA. Normally distributed continuous variables are presented as mean ± standard deviation, while the median and interquartile range represent non-normally distributed variables. A linear mixed model analyzed the recovery pattern over time among groups and interactions between CONUT and time for the subgroup that survived and completed the six-month follow-up. The repeated measures data were analyzed using only complete cases. The analysis was adjusted for confounders, including age, sex, National Institutes of Health Stroke Scale (NIHSS) score, hypertension, diabetes mellitus, coronary heart disease, atrial fibrillation, hyperlipidemia, smoking, alcohol consumption, heart, renal, liver, or malignant diseases. A Bonferroni post hoc analysis was utilized to determine differences between groups at each time point. A Kaplan–Meier methodology was used to construct survival curves, with log-rank tests evaluating their differences post-adjustment for confounding factors such as age, NIHSS score, hypertension, diabetes mellitus, coronary heart disease, atrial fibrillation, hyperlipidemia, smoking, alcohol consumption, heart, renal, liver, or malignant diseases. The patients were further divided into two subgroups based on their initial NIHSS scores [29]: mild stroke (NIHSS scores 0–4) and moderate/severe stroke (NIHSS scores ≥ 5), to ascertain if outcomes varied with stroke severity. All statistical analyses were conducted using SAS version 9.3 (SAS Institute Inc., Cary, NC, USA), with statistical significance established at *p* < 0.05.

## 3. Results

### 3.1. Baseline Characteristics

**Figure 1:**
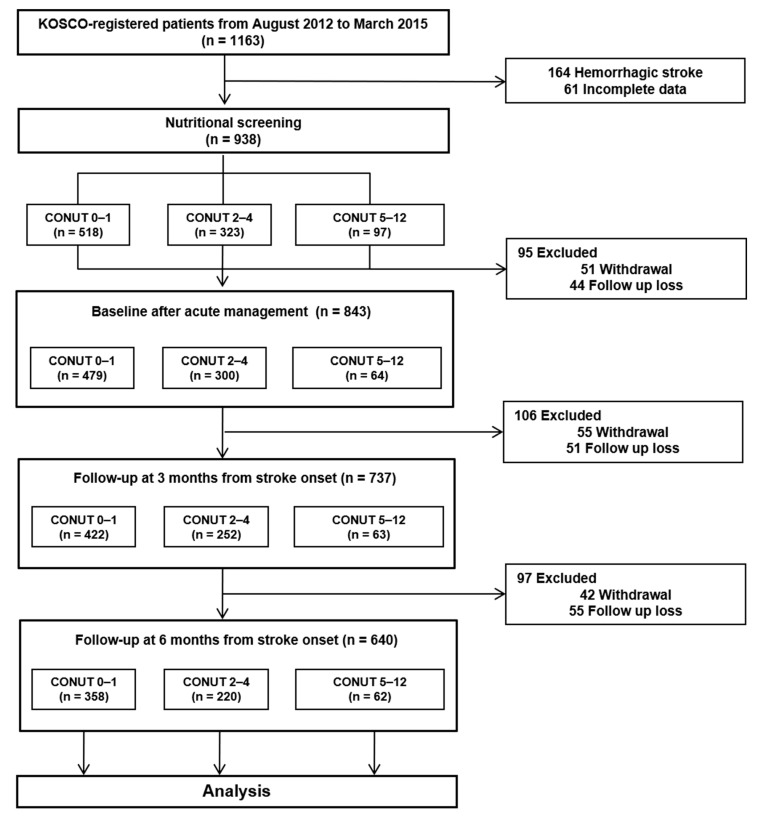
Flowchart of the study protocol.

A total of 1163 patients admitted at Severance Hospital, Yonsei University College of Medicine, gave their informed consent to participate in the study. Of these, 164 were excluded due to a diagnosis of hemorrhagic stroke. Furthermore, an additional 61 patients were excluded due to incomplete data or inability to assess their nutritional status immediately after admission. Among the 938 ischemic stroke patients who were analyzed, 150 experienced follow-up loss, and 148 withdrew from the study. The remaining 640 patients either completed the six-month follow-up or died during this period. Within this group, 358 (55.6%) were categorized into the CONUT 0–1 group, 220 (34.2%) into the CONUT 2–4 group, and 62 (9.6%) into the CONUT 5–12 group, respectively (Figure 1). No significant differences were observed in the baseline characteristics between patients who completed the six-month follow-up and those who did not (Supplementary Table S1). Upon admission, patients with high CONUT were notably older, had a lower BMI, and had higher NIHSS scores compared to those in the CONUT 0–1 group (all *p* < 0.05). No significant differences were observed in terms of sex, smoking history, alcohol consumption, other comorbidities, ischemic stroke subtype, stroke lesion location, or the time elapsed from onset to ER admission between the groups. The duration of acute neurological management was significantly longer for the higher CONUT group compared to the CONUT 0–1 group. Detailed baseline characteristics based on CONUT scores can be found in Table 1. Supplementary Table S2 presents the laboratory data of the two subgroups categorized by stroke severity.

### 3.2. Functional Outcomes

**Figure 2:**
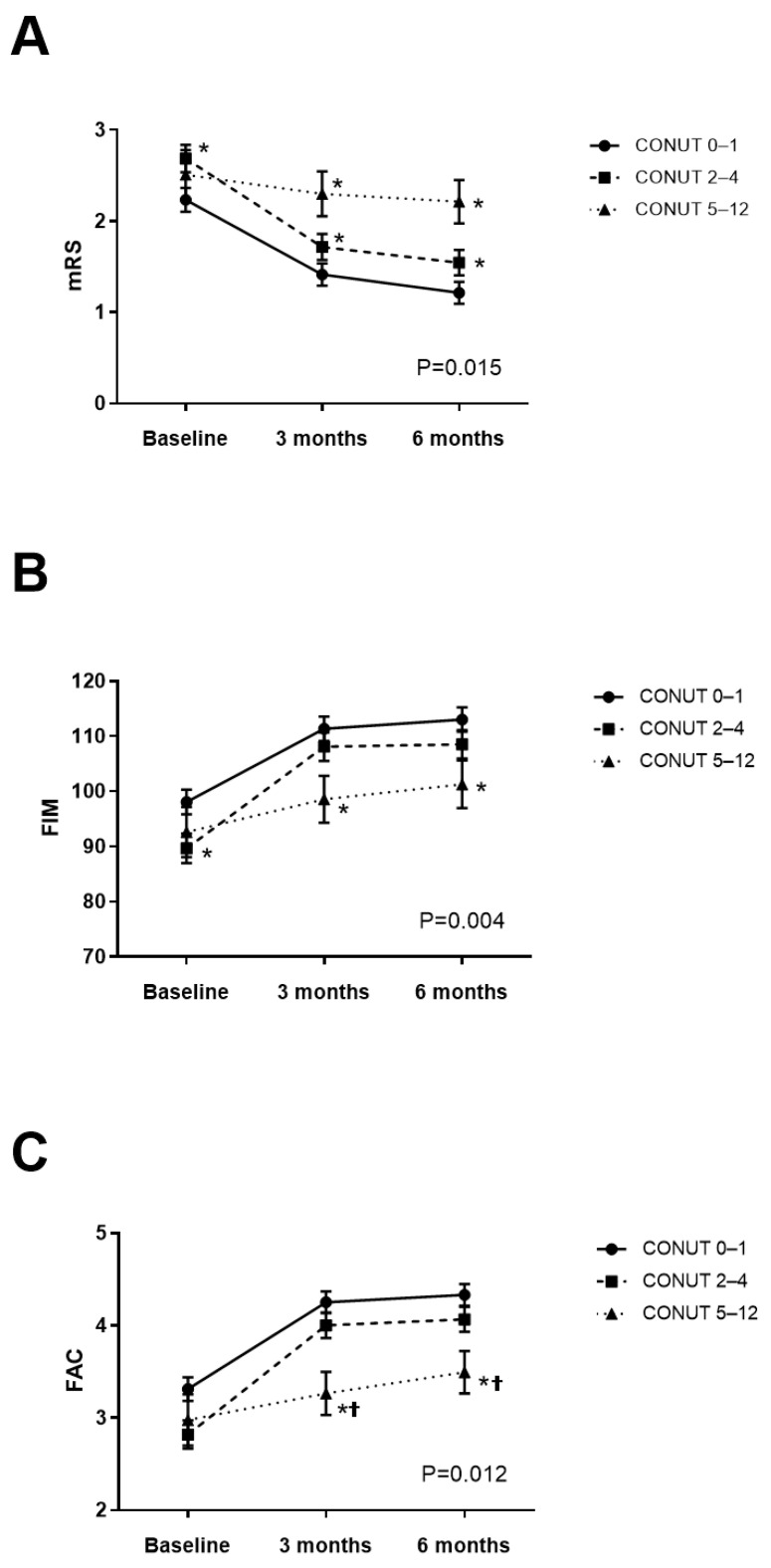
Changes in functional outcomes over the six months following stroke, categorized by CONUT scores: (**A**) mRS; (**B**) FIM; and (**C**) FAC. The analysis was adjusted for confounders, including age, sex, NIHSS score, hypertension, diabetes mellitus, coronary heart disease, atrial fibrillation, hyperlipidemia, smoking, alcohol consumption, heart, renal, liver, or malignant diseases. CONUT, Controlling Nutritional Status; mRS, modified Rankin Scale; FIM, Functional Independence Measure; FAC, Functional Ambulatory Category. * post hoc analysis, compared with CONUT 0–1 group, *p* < 0.05, † post hoc analysis, compared with CONUT 2–4 group, *p* < 0.05.

All groups, at three and six months post-stroke, showed significant improvement in mRS, FIM, and FAC scores regardless of their CONUT scores when compared to baseline values (all *p* < 0.01). However, the effects of the CONUT × time interaction on mRS, FIM, and FAC were significant after adjusting for age, initial stroke severity, stroke risk factors, and comorbidities (mRS: partial η^2^ = 0.008, effect size f = 0.090, *p* < 0.05; FIM: partial η^2^ = 0.012, effect size f = 0.110, *p* < 0.05; FAC: partial η^2^ = 0.011, effect size f = 0.105, *p* < 0.05, Figure 2), with a post hoc power exceeding 0.8, indicating adverse association between the CONUT scores and functional outcomes during the six months following a stroke. The post hoc analysis revealed that both the CONUT 2–4 and CONUT 5–12 groups had significantly higher mRS than the CONUT 0–1 group at three and six months (*p* < 0.05, Figure 2A). Furthermore, the CONUT 5–12 group exhibited significantly lower FIM than the CONUT 0–1 group (*p* < 0.05, Figure 2B) and significantly lower FAC scores than both CONUT 0–1 and CONUT 2–4 groups at three and six months (*p* < 0.05, Figure 2C).

In cases of mild stroke, significant effects of CONUT × time interactions on mRS and FAC were observed over the six months following stroke (*p* < 0.05, Table 2), although no significant effects were seen on FIM. The post hoc analysis showed that the CONUT 5–12 group demonstrated significantly higher mRS than both the CONUT 0–1 and CONUT 2–4 groups at both three and six months, significantly lower FAC than both CONUT 0–1 and CONUT 5–12 groups at 3 months, and significantly lower FAC than the CONUT 0–1 group at six months (*p* < 0.05). Conversely, no significant effects of the CONUT × time interaction on mRS, FIM, or FAC were found during the six months post-stroke in the moderate/severe stroke group (Table 2).

### 3.3. Survival Rate

**Figure 3:**
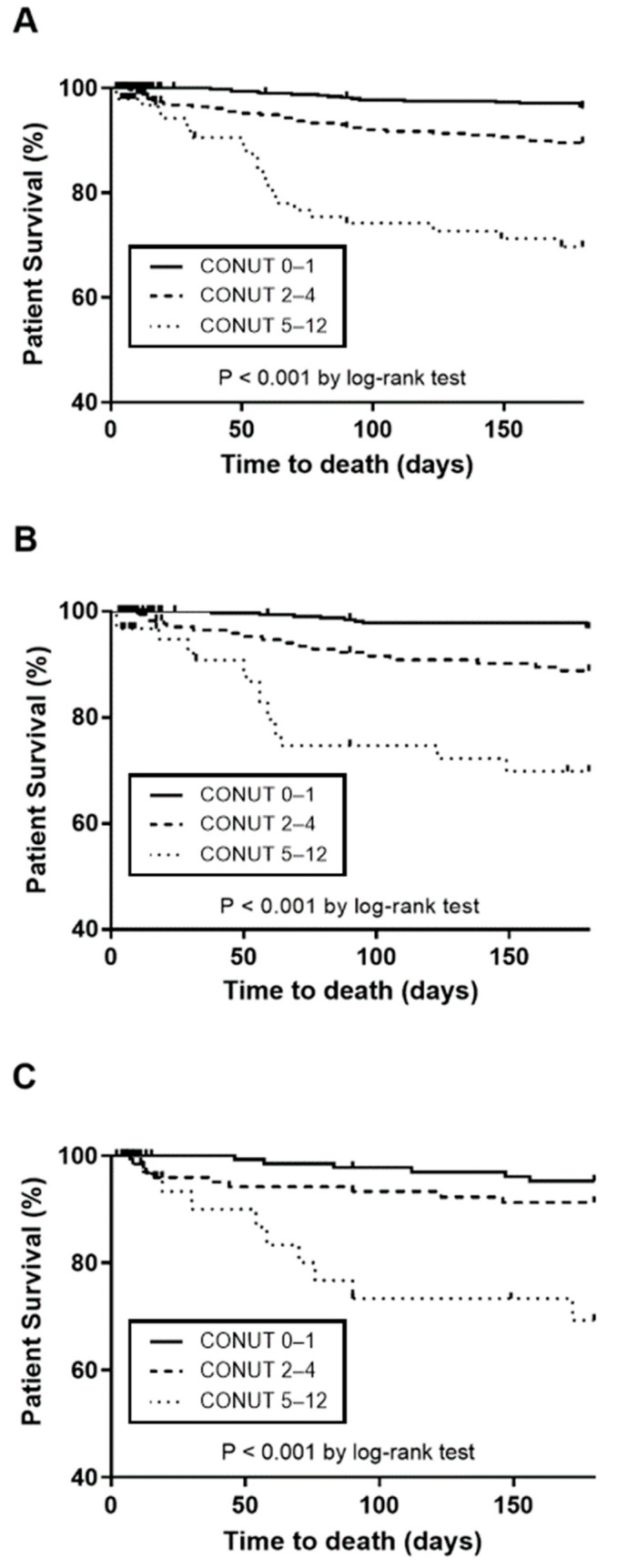
Comparison of Kaplan–Meier survival curves among different patient groups. (**A**) All patients; (**B**) patients with mild stroke; and (**C**) patients with moderate/severe stroke. The analysis was adjusted for confounders, including age, sex, NIHSS score, hypertension, diabetes mellitus, coronary heart disease, atrial fibrillation, hyperlipidemia, smoking, alcohol consumption, heart, renal, liver, or malignant diseases. CONUT, Controlling Nutritional Status.

The six-month survival rate was 96.4% in the CONUT 0–1 group, 89.8% in the CONUT 2–4 group, and only 69.7% in the CONUT 5–12 group post-stroke. The difference in survival rates among these groups was statistically significant over the six-month period following the stroke (log-rank test, *p* < 0.01, Figure 3A). Notably, the CONUT 5–12 group exhibited significantly poorer survival compared to both the CONUT 0–1 and CONUT 2–4 groups (*p* < 0.01). This reduced-survival trend in the CONUT 5–12 group, irrespective of the severity of the stroke, persisted when compared to the other two groups (*p* < 0.01, Figure 3B,C). The Kaplan–Meier survival curves illustrating these differences are presented in Figure 3.

### 3.4. Complications

The CONUT 5–12 group experienced a higher overall incidence of complications during hospitalization after stroke compared to both the CONUT 0–1 and CONUT 5–12 groups. Specifically, the incidence of pneumonia, urinary tract infections, pressure sores, falling injuries, and fractures was significantly higher in the CONUT 5–12 group than in both the CONUT 0–1 and CONUT 2–4 groups (all *p* < 0.01, Table 3).

## 4. Discussion

This study demonstrated that the initial CONUT score can predict functional outcomes, complications, and survival rates in patients experiencing their first-ever ischemic stroke, even after adjusting for confounding factors in a large-scale cohort study. Notably, the CONUT 5–12 group exhibited a higher incidence of complications during hospitalization, poorer functional outcomes, and a lower survival rate during the six months post-stroke compared to the other two groups.

In the current investigation, the CONUT score was employed to evaluate the nutritional status. The CONUT serves as an objective tool that can be efficiently and reliably measured using a blood sample [21]. Each of its three components assesses a different aspect of nutritional status: albumin for impaired protein metabolism, total cholesterol levels for lipid metabolism, and lymphocyte count for immune function. Previous research has shown that the number of patients at risk of malnutrition identified by the CONUT was greater than that identified by the Nutritional Risk Screening Tool 2002 (NRS-2002), suggesting that the CONUT may offer superior sensitivity [21]. In research conducted by Naito et al. [20], a high CONUT score was associated with unfavorable three-month outcomes, while low GNRI and anemia were not significantly correlated with outcomes. Utilizing the CONUT score is a convenient and cost-effective approach for comprehensive malnutritional risk assessment and poor outcome prediction in ischemic stroke.

This study offered a comprehensive overview of the functional outcomes in ischemic stroke patients, focusing on initial baseline CONUT scores. The CONUT score upon admission was found to be significantly associated with the functional recovery observed in this research. The general pattern of functional recovery demonstrated rapid and substantial improvements up to 3 months post-stroke, followed by a more gradual progression up to six months in both the CONUT 0–1 and CONUT 2–4 groups, which aligns with findings from previous research [30]. In contrast, the CONUT 5–12 group exhibited slower and consequently poorer recovery, even after adjusting for confounding factors.

The results of this study are in agreement with earlier research on the correlation between nutrition and functional outcomes post-stroke [10,11,21]. The Feed Or Ordinary Diet (FOOD) trial has shown that malnutrition immediately following a stroke is linked with unfavorable outcomes (mRS 3 to 5) six months later [10]. Furthermore, Shen et al. [11] demonstrated that malnutrition on admission predicts poor independence (Barthel Index < 75) up to six months post-stroke. In their study, although older age, higher initial NIHSS scores, and malnutrition upon admission were all significant predictors of poor outcomes, malnutrition had the highest odds ratio among these factors. A recent study involving 264 patients assessed nutritional status using the CONUT score and found that high CONUT score (CONUT 5–12) at admission is associated with poor outcomes (mRS 3–6) at three months after an ischemic stroke [21]. These findings, along with results from the current study and previous research, reinforce the evidence supporting the link between poor nutritional status and adverse functional outcomes following ischemic stroke.

However, other studies have presented varied findings about the relationship between nutritional status and functional outcomes [7,12]. Davis et al. [7] reported a significant, albeit unadjusted, association between malnutrition and poor one-month outcomes (MRS ≥ 3) using the Subjective Global Assessment (SGA). Yet, this association was not statistically significant after adjustments. The small sample size and the subjective nature of the evaluation tool could account for the differences in results compared to this study. Despite this, the importance of nutritional status as a modifiable factor was underscored, given the notable effect size of the associations, even though they did not reach statistical significance in the adjusted data. Pellicane et al. [12] have also reported that FIM efficiency is not associated with prealbumin levels, protein intake, or caloric intake, suggesting no clear relationship between nutritional status or intake and functional outcomes. It is important to note that prealbumin, with a rapid turnover and a half-life of 2–3 days, can serve as a sensitive indicator of recent nutritional intake, whereas indicators used in CONUT reflect longer-term nutritional status. Differences in the tools for assessing nutrition, the timing of the assessments, and the duration of follow-up might explain the variances observed between this study and previous research.

In this study, the association between the CONUT score and functional recovery was notably more significant for patients with mild stroke compared to those with moderate or severe stroke. This observation aligns with the findings from Qin et al., who reported that malnutrition at admission independently contributed to poor functional prognosis (mRS 3–6) at three months for patients with mild stroke, but not significantly for severe stroke patients [31]. The reasons for this disparity remain unclear. It is hypothesized that outcomes in patients with severe stroke, who often sustain substantial brain damage, may be more heavily influenced by factors other than nutritional status at admission. Another plausible explanation is that patients with severe stroke, due to their generally reduced mobility, are more prone to malnutrition [32]. Such patients may undergo nutritional changes during hospitalization, diminishing the impact of malnutrition at admission on their overall functional recovery. It is also notable that many patients with severe stroke reside in institutional settings where physical inactivity is common. This inactivity can lead to missed opportunities for adequate food intake, compounded by the need for assistance with daily activities, including eating. Recent studies have highlighted the positive outcomes associated with nutritional support for acute stroke patients [33,34]. Thus, early identification of malnutrition and subsequent nutritional interventions are crucial to optimizing outcomes for malnourished patients. Further research is needed to elucidate the mechanisms underlying the relationship between nutritional status, stroke severity, and functional recovery.

In this study, the CONUT score was found to be significantly associated with survival rates following an ischemic stroke. Patients categorized with CONUT 5–12 exhibited lower survival rates compared to those with CONUT 0–1 or CONUT 2–4 during the six months post-stroke. This observation aligns with previous research findings [5,10,35]. Lim et al., conducting a study in Singapore that evaluated the nutritional status of patients with a variety of medical and surgical conditions using the SGA, reported that malnutrition was associated with a fourfold increase in the risk of mortality at one-year follow-up [5]. Similarly, in the FOOD trial [10], which focused on stroke patients, 37% of undernourished patients versus 20% of patients with normal nutritional status died within six months. Furthermore, Gomes et al. [35] reported that over 40% of the patients identified as high risk for malnutrition using the Malnutrition Universal Screening Tool died after the stroke, compared to less than 6% of the patients with normal nutritional status.

This study also documented a higher incidence of pneumonia, urinary tract infections, pressure ulcers, falls, and fractures, along with a longer duration of acute management in patients with high CONUT score, findings that are consistent with those of previous studies [5,29]. Malnutrition is known to compromise immunity, rendering patients more susceptible to infections [36]. Additionally, malnutrition can hinder wound healing, decrease bone mass, and increase the risk of fractures [37,38]. These complications noted in the study could partially account for the observed differences in functional recovery and survival rates among the different groups.

Additional mechanisms can be proposed for how nutritional status upon admission may link with long-term functional recovery, complications, and survival after a stroke. It has been demonstrated that malnutrition, inflammation, and oxidative stress, collectively encompassed by the “malnutrition–inflammation complex syndrome” (MICS), are predictive of mortality in individuals with chronic kidney disease [39,40]. MICS is characterized by low BMI, hypocholesterolemia, hypoalbuminemia, and hypohomocysteinemia [39]. In this study, the CONUT 5–12 group exhibited the highest levels of CRP (a marker of malnutrition and inflammation) yet the lowest BMI among the evaluated groups. Additionally, stroke may be considered an inflammatory disease, as the inflammatory process is initiated at the onset of stroke. Thus, there may be an association between stroke and MICS. While only a limited number of studies have explored the pathophysiology of MICS in ischemic stroke, there is substantial evidence indicating that inflammatory cytokines, such as tumor necrosis factor, can contribute to tissue degradation and facilitate the loss of skeletal muscle [41,42]. Muscle mass is directly linked to muscle strength and cardiopulmonary fitness [43]. Consequently, malnutrition may impede muscle strengthening and functional recovery post-stroke. The condition described as “stroke-related sarcopenia”, which is marked by muscle mass loss, muscle fatty infiltration, and skeletal muscle atrophy in the affected limbs, is induced by malnutrition. This can result in restricted mobility and poorer rehabilitation outcomes [44]. Moreover, the vicious cycle between hypomobility and sarcopenia can further aggravate malnutrition [32]. These mechanisms relate to the concept of the ‘obesity paradox’, wherein obese patients might benefit from an energy reservoir in catabolic states, potentially leading to a better prognosis [45]. Therefore, it is critical to assess baseline nutritional status to facilitate the early detection and correction of malnutrition, aiming to optimize the functional recovery and survival rates of stroke patients.

## 5. Limitation

This study is subject to several limitations. Firstly, the data were sourced from a single center and only included patients who completed the six-month follow-up, which may limit the generalizability of the findings despite the inclusion of a sizable number of subjects. This may also introduce the potential for selection bias. Nonetheless, it was verified that there were no significant differences in the baseline characteristics between the participants who completed the six-month follow-up and those who did not. Secondly, the CONUT score in this study was used as a screening tool rather than a comprehensive assessment, which lacked inclusion of subjective indicators or anthropometric measurements. It is crucial to highlight that, as of now, no universally accepted gold standard for identifying malnutrition in stroke patients exists. Future studies should aim to compare the CONUT score with other nutritional assessment tools like the prognostic nutritional index, mini nutritional assessment, etc. Despite the inherent limitations of the screening tool used, it is important to emphasize that the CONUT score offers a straightforward, quick, and dependable means of assessing protein reserves, caloric deficiency, and immune status. Thirdly, the study included relatively few patients with moderate to severe stroke, which impedes definitive conclusions about the variation in functional recovery based on stroke severity. Fourth, given the low incidence of post-stroke complications, the observed differences in complications carry the potential for type I or type II errors. Further research with a larger sample size is required to confirm these findings. Lastly, serial assessments of nutritional status or dietary intakes were not conducted. Further research is essential to elucidate the detailed mechanisms involved in the pathophysiological pathways and to develop innovative therapeutic strategies to enhance functional recovery in malnourished stroke patients.

## 6. Conclusions

This study demonstrated that initial CONUT score at admission predicted complications, impaired functional recovery, and increased mortality. Therefore, CONUT scores could be recommended as an integral part of the early clinical nutritional assessment following an acute ischemic stroke.

## Figures and Tables

**Table 1:** General characteristics of patients according to CONUT score.

Variable	CONUT 0–1 (n = 358)	CONUT 2–4 (n = 220)	CONUT 5–12 (n = 62)	*p*-Value
Age, years	63.90 ± 13.02	66.73 ± 13.83	69.23 ± 12.66	0.003
Male, n (%)	207 (57.8)	133 (60.5)	38 (61.3)	0.767
Body mass index (kg/m^2^)	24.46 ± 3.66	23.71 ± 3.82	21.58 ± 3.11	<0.001
Smoking, n (%)	106 (29.6)	41 (18.6)	10 (16.1)	0.003
Alcohol, n (%)	162 (45.3)	82 (37.3)	28 (45.2)	0.153
Hypertension, n (%)	231 (64.5)	146 (66.4)	45 (72.6)	0.460
Diabetes mellitus, n (%)	89 (24.9)	69 (31.4)	15 (24.2)	0.202
Coronary heart disease, n (%)	44 (12.3)	39 (17.7)	11 (17.7)	0.155
Atrial fibrillation, n (%)	53 (14.8)	49 (22.3)	13 (21.0)	0.062
Hyperlipidemia, n (%)	49 (13.7)	26 (11.8)	6 (9.7)	0.612
Congestive heart failure, n (%)	8 (2.2)	7 (3.2)	4 (6.5)	0.191
Renal disease, n (%)	3 (0.8)	12 (5.5)	2 (3.2)	0.003
Liver disease, n (%)	7 (2.0)	5 (2.3)	3 (4.8)	0.382
Malignant disease, n (%)	10 (2.8)	16 (7.3)	1 (1.6)	0.019
Ischemic stroke subtype, n (%)				0.053
Large-artery atherosclerosis	192 (53.6)	140 (63.6)	42 (67.7)	
Small-artery occlusion	133 (37.2)	65 (29.5)	12 (19.4)	
Cardioembolism	15 (4.2)	8 (3.6)	5 (8.1)	
Other determined	12 (3.4)	3 (1.4)	2 (3.2)	
Undetermined	6 (1.7)	4 (1.8)	1 (1.6)	
Lesion site, n (%)				0.296
Cortical	100 (27.9)	77 (35.0)	21 (33.9)	
Subcortical	178 (49.7)	86 (39.1)	28 (45.2)	
Brainstem	55 (15.4)	36 (16.4)	8 (12.9)	
Multiple	25 (7.0)	21 (9.5)	5 (8.1)	
Thrombolytic therapy, n (%)	32 (8.9)	22 (10.0)	6 (9.7)	0.910
Endovascular therapy, n (%)	5 (1.4)	2 (0.9)	1 (1.6)	0.845
NIHSS	2.0 (1.0–5.0)	3.0 (1.0–7.0)	4.0 (2.0–7.25)	0.008
Time to admission at hospital, h	10.87 (2.99–33.90)	11.82 (2.60–33.91)	10.64 (1.00–34.23)	0.588
Duration of acute neurological management, days	6.00 (5.00–9.00)	8.00 (6.00–12.00)	10.00 (7.00–20.50)	<0.001
Serum albumin, g/L	4.19 ± 0.32	3.98 ± 0.40	3.16 ± 0.44	<0.001
Total cholesterol, mg/dL	200.07 ± 43.32	162.69 ± 36.90	133.69 ± 42.21	<0.001
Total lymphocyte count, /mm^3^	2325.87 ± 759.19	1478.68 ± 758.25	931.77 ± 444.98	<0.001
*C*-reactive protein, mg/L	8.19 ± 21.25	17.43 ± 30.71	46.77 ± 57.12	<0.001
CONUT score	0.46 ± 0.50	2.68 ± 0.76	6.74 ± 2.01	<0.001

Values are expressed as mean ± standard deviation for normally distributed variables or median (interquartile range) for non-normally distributed variables. CONUT, Controlling Nutritional Status; NIHSS, National Institutes of Health Stroke Scale.

**Table 2:** Functional outcomes at baseline, three-month, and six-month follow-up for mRS, FIM, and FAC, according to CONUT score.

Nutritional Status	CONUT 0–1	CONUT 2–4	CONUT 5–12	*p*-Value
Mild stroke (n = 385)				
mRS				
CONUT × time interaction				0.014
Baseline	1.68 ± 0.14	1.99 ± 0.17	1.96 ± 0.31	0.148
3 months	0.93 ± 0.13	1.16 ± 0.16	2.04 ± 0.27 *†	<0.001
6 months	0.73 ± 0.13	1.03.± 0.15	1.93 ± 0.26 *†	<0.001
FIM				
CONUT × time interaction				0.121
Baseline	109.22 ± 2.23	104.24 ± 2.81	103.45 ± 4.78	0.109
3 months	119.94 ± 2.10	116.48 ± 2.55	105.2 ± 4.13 *†	<0.001
6 months	120.93 ± 2.12	117.17 ± 2.60	107.69 ± 4.30 *	0.004
FAC				
CONUT × time interaction				0.037
Baseline	3.88 ± 0.13	3.59 ± 0.17	3.44 ± 0.31	0.129
3 months	4.64 ± 0.12	4.50 ± 0.14	3.52 ± 0.23 *†	<0.001
6 months	4.71 ± 0.11	4.50 ± 0.13	3.75 ± 0.23 *	<0.001
Moderate/severe stroke (n = 187)				
mRS				
CONUT × time interaction				0.708
Baseline	3.15 ± 0.27	3.83 ± 0.29 *	3.54 ± 0.51	0.040
3 months	2.16 ± 0.27	2.66 ± 0.28	2.83 ± 0.50	0.105
6 months	1.95 ± 0.26	2.43 ± 0.28	2.78 ± 0.49	0.063
FIM				
CONUT × time interaction				0.132
Baseline	75.97 ± 5.38	62.88 ± 5.71 *	66.63 ± 9.74	0.038
3 months	96.01 ± 5.34	89.71 ± 5.66	79.78 ± 9.59	0.152
6 months	99.58 ± 5.31	89.94 ± 5.63	81.95 ± 9.63	0.054
FAC				
CONUT × time interaction				0.708
Baseline	2.20 ± 0.29	1.49 ± 0.30 *	1.92 ± 0.55	0.046
3 months	3.60 ± 0.28	3.05 ± 0.30 *	2.56 ± 0.52 †	0.038
6 months	3.73 ± 0.28	3.22 ± 0.29	2.79 ± 0.51	0.053

Values are expressed as estimated mean ± standard deviation. The analysis was adjusted for confounders, including age, sex, NIHSS score, hypertension, diabetes mellitus, coronary heart disease, atrial fibrillation, hyperlipidemia, smoking, alcohol consumption, heart, renal, liver, or malignant diseases. CONUT, Controlling Nutritional Status; mRS, modified Rankin Scale; FIM, Functional Independence Measure; FAC, Functional Ambulatory Category. * post hoc analysis, compared with CONUT 0–1 group, *p* < 0.05, † post hoc analysis, compared with CONUT 2–4 group, *p* < 0.05.

**Table 3:** Post-stroke complications during hospitalization according to CONUT scores.

Complications	All	CONUT 0–1	CONUT 2–4	CONUT 5–12	*p* Value
Thromboembolic disease	11 (1.9)	6 (1.8)	4 (2.1)	1 (2.6)	0.914
Pneumonia	19 (3.3)	4 (1.2)	9 (4.7)	6 (15.8)	<0.001
Ventilatory insufficiency	2 (0.3)	2 (0.6)	-	-	0.509
Urinary tract infection	17 (3.0)	3 (0.9)	8 (4.2)	6 (15.8)	<0.001
Pressure sore	3 (0.5)	-	1 (0.5)	2 (5.3)	<0.001
Fall and injuries	1 (0.2)	-	-	1 (2.6)	0.001
Fracture	1 (0.2)	-	-	1 (2.6)	0.001
Complex regional pain syndrome	2 (0.3)	1 (0.3)	1 (0.5)	-	0.849
Central post-stroke pain syndrome	1 (0.2)	1 (0.3)	-	-	0.714

Values are expressed as number of patients (%). CONUT, Controlling Nutritional Status.

## Data Availability

Data will be made available upon reasonable request to the corresponding author due to legal and ethical reasons.

## Supplementary Materials

The following supporting information can be downloaded at: https://www.mdpi.com/article/10.3390/nu16203461/s1, Supplementary Table S1. General characteristics of the study cohort; Supplementary Table S2. Laboratory data according to CONUT score.
