# Supplementary material for: The Significance of an Initial Controlling Nutritional Status Score in Predicting the Functional Outcome, Complications, and Mortality in a First-Ever Ischemic Stroke
Source: Nutrients. 2024 Oct 12;16(20):3461. doi: 10.3390/nu16203461 (PMC11509889; doi:10.3390/nu16203461)
Supplement: Supplementary file 1 [file nutrients-16-03461-s001.zip › nutrients-3241876-supplementary.pdf]

Supplementary Table S1. General characteristics of the study cohort

Variable	Total (n = 938)	Enrollment (n = 640)	Drop out (n = 298)	P-value
Age, years	65.80 ± 13.26	65.39 ± 13.37	64.57 ± 12.67	0.375
Men, n (%)	560 (59.7)	378 (59.1)	182 (61.1)	0.559
Body mass index (kg/m²)	23.89 ± 3.61	23.92 ± 3.76	23.99 ± 3.15	0.762
Smoking, n (%)	243 (25.9)	157 (24.5)	86 (28.9)	0.159
Alcohol, n (%)	414 (44.1)	272 (42.5)	142 (47.7)	0.139
Hypertension, n (%)	620 (66.1)	422 (65.9)	198 (66.4)	0.879
Diabetes mellitus, n (%)	264 (28.1)	173 (27.0)	91 (30.5)	0.266
Coronary heart disease, n (%)	146 (15.6)	94 (14.7)	52 (17.4)	0.277
Atrial fibrillation, n (%)	157 (16.7)	115 (18.0)	42 (14.1)	0.139
Hyperlipidemia, n (%)	122 (13.0)	81 (12.7)	41 (13.8)	0.640
Congestive heart failure, n (%)	30 (3.2)	19 (3.0)	11 (3.7)	0.558
Renal disease, n (%)	23 (2.5)	17 (2.7)	6 (2.0)	0.553
Liver disease, n (%)	17 (1.8)	15 (2.3)	2 (0.7)	0.074
Malignant disease, n (%)	33 (3.5)	27 (4.2)	6 (2.0)	0.088
Ischemic stroke subtype, n (%)				0.442
Large–artery atherosclerosis	548 (58.4)	374 (58.4)	174 (58.4)	
Small-artery occlusion	318 (33.9)	210 (32.8)	108 (36.2)	
Cardioembolism	36 (3.8)	28 (4.4)	8 (2.7)	
Other determined	22 (2.3)	17 (2.7)	5 (1.7)	
Undetermined	14 (1.5)	11 (1.7)	3 (1.0)	
Affected lesion, n (%)				0.697
Cortical	283 (30.2)	198 (30.9)	85 (28.5)	
Subcortical	429 (45.7)	292 (45.6)	137 (46.0)	
Brainstem	145 (15.5)	99 (15.5)	46 (15.4)	
Multiple level	81 (8.6)	51 (8.0)	30 (10.1)	
Thrombolytic therapy, n (%)	84 (9.0)	60 (9.4)	24 (8.1)	0.509
Endovascular therapy, n (%)	11 (1.2)	8 (1.3)	3 (1.0)	0.747
NIHSS	4.75 ± 5.40	4.72 ± 5.42	4.87 ± 5.35	0.679

Time to admission at hospital, h	22.39 ± 28.20	23.58 ± 28.78	20.56 ± 26.62	0.115
Duration of acute neurologic management, days	9.57 ± 10.44	9.82 ± 9.87	9.19 ± 12.14	0.395

Values are expressed as mean ± standard deviation for normally distributed variables, or median (interquartile range) for non-normally distributed variables. CONUT, Controlling Nutritional Status; NIHSS, National Institutes of Health Stroke Scale.

Supplementary Table S2. Laboratory data according to CONUT score

Variable	CONUT 0-1	CONUT 2-4	CONUT 5-12	P-value
Mild stroke (n = 385)				
Serum albumin, g/L	4.23 ±0.31	4.03 ±0.39	3.22 ±0.49	< 0.001
Total cholesterol, mg/dL	202.00 ±45.31	165.93 ±37.87	142.25 ±55.69	< 0.001
Total lymphocyte count, /mm³	2340.65 ±753.24	1488.05 ±652.25	999.17 ±483.58	< 0.001
C-reactive protein, mg/L	5.09 ±18.65	4.75 ±11.04	41.05 ±47.55	< 0.001
CONUT score	0.42 ±0.50	2.60 ±0.71	6.67 ±2.62	< 0.001
Moderate to severe stroke (n = 187)				
Serum albumin, g/L	4.12 ±0.30	3.99 ±0.36	3.19 ±0.46	< 0.001
Total cholesterol, mg/dL	197.09 ±39.22	157.15±35.32	132.29±41.87	< 0.001
Total lymphocyte count, /mm³	2323.72 ±804.05	1463.29 ±877.95	916.43 ±473.59	< 0.001
C-reactive protein, mg/L	12.28 ±22.25	19.63 ±27.40	53.00±69.84	< 0.001
CONUT score	0.55±0.50	2.71±0.75	6.86±1.51	< 0.001

Values are expressed as mean ± standard deviation. CONUT, Controlling Nutritional Status
